# Reviews

**Published:** 1888-10

**Authors:** 


### REVIEWS.

The Medical and Surgical History of the War of the Rebellion. Part 3, Vol. I., Medical History. Being the Third Medical Volume. Prepared under the Direction of the Surgeon General U. S. Army. By Major and Surgeon U. S. Army, Charles Smart. Washington Government Printing Office.

The preservation of the records of our great civil war cannot fail to possess an absorbing interest for those of a generation to whom the echoes of the mighty struggle have come down the avenues of time with comparative in distinctness, but when in addition to the historical value of such a record, we find added the useful feature of scientific information, its advantage and interest become vastly enhanced. Such is the character of the volume just issued by the Government Press in Washington, and history and science alike owe a lasting debt of gratitude to Dr. Smart for his invaluable contribution to the medical lore of the rebellion. The undertaking was a stupendous one and nothing short of indomitable energy and inexhaustible patience could have brought it to a successful consummation, Let the reader imagine 1,000 closely printed pages of clinical and statistical information, culled from every known source in the Northern and Southern States, and he can form an idea of the labor which the compilation of such a volume involved. The chief difficulty, no doubt, which beset the learned author's endeavor to lend interest to this chaotic mass of historical and scientific data was to reduce them to suitable order. It must have appeared to him a truly herculean task, and only a logical and well disciplined mind could have avoided ever-threatening confusion. Any attempt at even the most compendious resumè of the work would lead us beyond the limits within which this line of review must be confined, so that we can only advert to a few facts, scattered here and there. The average reader of newspapers, recalling the heavy head lines in which the story of bloody battles and dreadful carnage was recorded, will be surprised to learn that of the total number of deaths in the Northern armies, caused by the war (304,369) only 44,238 were caused by death wounds in the battle field while the appalling fatality of 186,216 resulted from disease. No more eloquent comment could there be on the inexperience in camp life and the ignorance of sanitary matters which existed in the early days of the rebellion, very nearly the same proportion of deaths from similar causes marked the career of the Confederate armies. Swamps and continued fevers contracted through exposure and a defective commissariat were the chief factors in producing death. The pathology of these diseases was closely studied throughout the war by many competent observers whose researches have been embodied in his work by Dr. Smart, and which may be regarded as a valuable contribution to medical literature. Many excellent photo-reliefs and chromolithographs illustrate the various morbid condition of the intestinal tract during fever. It is the carefully tabulated statistics on an almost endless variety of subjects that impart the chief value to this magnificent volume and fully attest the indefatigable industry of its author. C. M. O'L.

Thiermedizinische Vortrage.—This publication, edited by Dr. Georg Schneidemuhl in Halle, is devoted the progress made in veterinary science, each number being a monograph on the subject treated. Three numbers have already been published on the following subjects. The Swine Disease, by Prof. E. Hess of Bern; Progress on the Science of

Digestion, by Prosector Edelmann of Dresden; and Progress in the Treatment of Wounds, by Dr. Muller of Dresden. The coming number will be on the progress in drugs and the science of making up prescriptions. The numbers are issued monthly. Price, 12 marks per year. C.

University Medical Magazine.—The first number of this magazine has just been published. It is edited under the auspices of the Faculty of Medicine and the Alumni of the University of Pennsylvania, composed of such eminent men as Pepper, Agnew, Huidekoper, Osler, Goodell, etc., names well known in the scientific world, and which are of themselves a guarantee of success to the undertaking.

The matter of the Journal will be entirely original, no "scissors" to be used.

The articles of the present number are "Practical Observations on Senile Hypertrophy of the Prostrate Gland," by D. H. Agnew, M.D., L.L.D. "A Year's Work in Orphorectomy," by William Goddell, A.M., M.D. "Thoughts Concerning the Symptomatology of Insanity," by H. C. Wood, M.D., L.L.D.; "Cardiocentesis," by Wm. Pepper, M.D., L.L.D. "Minor Difficulties in the Diagnosis of Hernias," by J. W. White, M.D., etc.

The Magazine is published by Dr. A. L. Hummel, 224 South 16th Street, Philadelphia.

